# Stages of Gut Development as a Useful Tool to Prevent Gut Alterations in Piglets

**DOI:** 10.3390/ani11051412

**Published:** 2021-05-14

**Authors:** Silvia Clotilde Modina, Lucia Aidos, Raffaella Rossi, Paola Pocar, Carlo Corino, Alessia Di Giancamillo

**Affiliations:** Dipartimento di Medicina Veterinaria, Università degli Studi di Milano, Via dell’Università 6, 26900 Lodi, Italy; silvia.modina@unimi.it (S.C.M.); lucia_aidos@hotmail.com (L.A.); raffaella.rossi@unimi.it (R.R.); paola.pocar@unimi.it (P.P.); carlo.corino@unimi.it (C.C.)

**Keywords:** gut development, cell turnover, morpho functional activity, piglets

## Abstract

**Simple Summary:**

Intensive growth and development of the piglet’s small intestine is a process that is faster than growth of the whole organism; moreover, it begins early in the prenatal period, accelerating soon after birth and continuing into the post-weaning phase. In fact, during gestation, the gut experiences important morpho-functional changes facilitated by hormones, growth factors, and luminal products. All of these changes are essential to ensure a correct gut function in order to protect animal health and welfare, to ensure optimal productive and reproductive performance, and reduce antimicrobial use.

**Abstract:**

During the prenatal, neonatal, and weaning periods, the porcine gastrointestinal tract undergoes several morpho-functional, changes together with substantial modification of the microbial ecosystem. Modifications of the overall structure of the small intestine also occur, as well as a rapid increase of the volume, mainly in the last period of gestation: intestinal villi, starting from jejunum, appears shortly before the sixth week of gestation, and towards the end of the third month, epithelial cells diversify into enterocytes, goblet cells, endocrine, and Paneth cells. Moreover, in the neonatal period, colostrum induces an increase in intestinal weight, absorptive area, and brush border enzyme activities: intestine doubles its weight and increases the length by 30% within three days of birth. During weaning, intestinal environment modifies drastically due to a replacement of highly digestible sow milk by solid feed: profound changes in histological parameters and enzymatic activity are associated with the weaning period, such as the atrophy of the villi and consequent restorative hypertrophy of the crypts. All these modifications are the result of a delicate and precise balance between the proliferation and the death of the cells that form the intestinal mucosa (i.e., mitosis and apoptosis) and the health conditions of the piglet. An in-depth knowledge of these phenomena and of how they can interfere with the correct intestinal function can represent a valid support to predict strategies to improve gut health in the long-term and to prevent weaning gut alterations; thus, reducing antimicrobial use.

## 1. Gastrointestinal Tract (Git) Development: A Stressful Moment for Piglets

There are many stressful events that affect the health and growth of piglets from birth to weaning: among these, the development of the gastrointestinal tract (GIT) is a complex and delicate process, which begins in the prenatal phase and then continues after birth.

**Figure 1:**
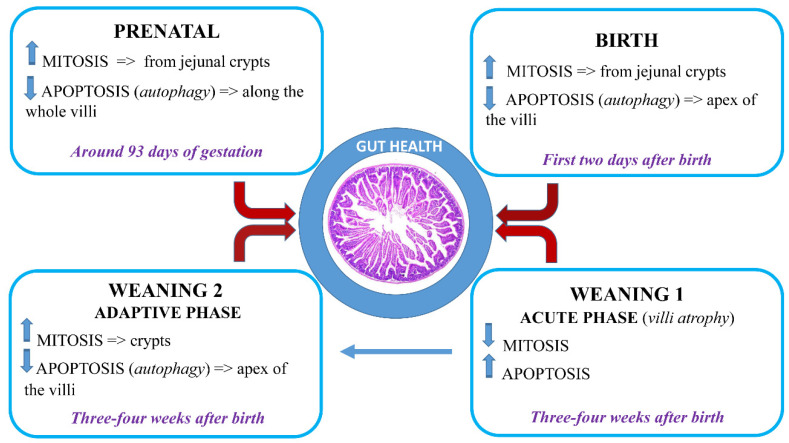
Timing of cellular turnover during gut development: apoptosis and mitosis.

The most important factor that can influence the structure and functions of GIT is diet: in the first postnatal period, the bioactive substances present in the colostrum and in the sow’s milk are essential for proper development and growth; their properties modulate cell physiology, such as the turnover of apoptosis and mitosis [1]. In that way, they also influence the development of GIT, allowing the adaptation of animals to solid feed (Figure 1). On the contrary, the weaning phase is related not only to the diet, but also to the proper development of the intestinal microflora.

Appropriate changes of the anatomical structure of the intestinal mucosa and its enzymatic activities are indispensable to allow to GIT to adapt to diet, as already previously described in pig during weaning [1,2].

This review will focus on the gut morphological development and growth in a wider period, from preterm to weaning. The aim is to provide useful tools to evaluate (predict) new strategies to improve gut homeostasis at the physiological, immunological, and microbial level in the long-term, and to prevent weaning gut alterations; thus, reducing antimicrobial use.

## 2. Gut Anatomy in Brief

### Focus on the Small Intestine

The small intestine is anatomically divided into three regions whose proportions are as follows: duodenum, which in adult pig represents about 4–4.5%, jejunum, about 88–91% and finally ileum, about 4–5% [3]. The newborn has similar proportions to those of the adult, even if the differentiation between jejunum and ileum is not well defined. Although in adults, these three traits have morphological characteristics that clearly distinguish them, they also share many common features as they are all organized in four basic layers: the tunica serosa, the tunica muscularis, the tunica submucosa, and the tunica mucosa. The serosa is the outer layer and morphologically consists of an epithelium lining on connective tissue, which contains blood vessels and nerves, and which eventually forms the mesentery. The tunica muscularis is organized with two distinct layers of muscle fibers; an outer layer of longitudinal fibers and an inner layer of circular fibers, which both are involved in GIT motility. The submucosa is made up of a layer of connective tissue that contains blood and lymphatic vessels and nerves.

A special focus should be done on the submucosa of the duodenum, because it contains the Brunner’s glands, which are specialized to produce alkaline secretion containing bicarbonate, in order to protect the duodenal mucosa from the acidic content of the chyme coming from the stomach; in addition, this secretion stimulates the activation of intestinal enzymes by creating an alkaline environment and lubricating its walls. Nutrition, nervous and/or hormonal stimuli and reflexes increase the secretory activity of Brunner’s glands [4].

The inner layer, the mucosa, is the one that characterizes the morpho-functional activity of each individual trait of the intestine and it is made up of three sublayers: the *muscularis mucosa*, the *lamina propria* and the epithelium. The *muscularis mucosa* is a thin layer of muscle fibers that divides the submucosa from the mucosa and that contributes to form the transient intestinal folds. The *lamina propria* is made up of connective tissue that contains blood vessels, neurons, lymphocytes and, in the ileum, it also contains lymph nodes called Peyer’s patches; *the lamina propria* has the duty of sustaining the avascular epithelial layer. This latter consists of a single layer of epithelial cells, which covers the luminal surface of the intestine. 

The mucosa as a whole, folds back to form finger-like projections called villi, and at the base of these ones, the Crypts of Lieberkühn, also known as intestinal glands, which are moat-like invaginations.

Villi increase the work surface by at least five times compared to a flat surface of the same size. Furthermore, the luminal surface of the enterocytes has got microvilli at the level of the apical membrane (also called brush border), which further increase the absorptive surface by 15–40 times, simulating the observed system of the villi folds. Microvilli are placed in a gelatinous layer of glycoproteins, the glycocalyx, and have digestive enzymes. The villi morphology changes according to the intestinal tracts and this trend reflects their different functions: the length increases from the duodenum to the mid-jejunum and then decreases again towards the distal ileum. Similarly, the crypts also change in size and composition along the intestine: they are deeper in the duodenum and jejunum and less deep in the ileum. [5].

If we look at the epithelial layer more closely, we notice that there are three types of cells lining on the villus surface: enterocytes, goblet cells, and enteroendocrine cells (piglet gut barrier reviewed by [6]). Enterocytes are the most abundant group of villi cells, about 94%, goblet cells are about 5% and endocrine cells about 1%. All of these cell types originate from stem cells located at the base of the crypts [7]. Enterocytes get mature while they migrate from the base to the tip of the villi; their enzymatic activity, which reflects the digestive function, begins when the enterocytes reach the basal third of the villi axis, while their absorption function begins when enterocytes reach the medium-high level and spreads its maximum when the cells reach the top of the villi. Obviously, the enterocytes present on the surface of the villi are continuously renewed.

The goblet cells are secreting cells: specifically, they secrete viscous mucus and are located between the enterocytes. They increase in number from the proximal portion of the jejunum to the distal one of the ileum. Goblet cells secrete relentlessly viscous mucus and their basal activity increases when the cells come in contact with a secretagogue substance. Mucus production can be altered by several agents: cholinergic agents, neuropeptides, hormones, and toxins for example. Furthermore, nutrients are capable of influencing the thickness and production of mucus (e.g., milk peptides). In addition, the enterocytes that organize the mucosa can also have secreting activity contributing to produce, with the mucus, the intestinal juice: a young pig of about 70 kg, produces approximately 6 L per day [3]. The third cell type, the enteroendocrine cells, produce hormones to regulate the functionality of the gastrointestinal tract [8].

Interesting, recent works suggest the presence in the GIT of pig of another type of cells, the Paneth cells, which have been described extensively in other species [9]. Reference [10] observed these cells, located adjacent to stem cells at the bottom of the crypt of the ileum, and [11] observed Paneth cell-like cells in the gut of piglet from newborn to weaned stages in the same localization. The exact function is unknown, but due to the presence of substances, such as lysozymes and defensins inside microgranules of the cytoplasm, they most likely contribute to maintenance of the gastrointestinal barrier. About the differentiation of Paneth cells, it would be possible to hypothesize that, as described for other species, they move back to the stem cell compartment to be interspersed with stem cells [12].

## 3. The Development of the Small Intestine during the Preterm Period

The rapid and massive growth of the small intestine in the pig, begins in the prenatal phase, just a few weeks before farrowing and it is by far the fastest growth of the whole organism [13], and there are tissue-specific activities of brush-border enzymes with low maltase and high lactase activities [14]. This may partly be due to increased ingestion of amniotic fluid, as suggested by [15].

The small intestine shows the presence of intestinal villi starting from jejunum, the site of maximum absorption, shortly before the sixth week of gestation and, towards the end of the third month, it is possible to observe the differentiation of the epithelial cells into the three cell types previously described. The morphological peculiarity of fetal enterocytes lies in the presence of large cytoplasmic vacuoles (endocytic activity) that, in the adult, will be replaced with cells without vacuoles. There are two types of vacuoles: (1) transport vacuoles, necessary for the transport of macromolecules from the intestinal lumen to the enterocytes; (2) digestive or lysosomal vacuoles, involved in the intracellular digestion of nutrients and pH control in the intestinal lumen [16]. The transport vacuoles are present throughout the small intestine in the first two days of birth and allow the bioactive compounds of the colostrum to cross the enterocytes without being modified, providing passive immunity; subsequently, there is a reduction of this transport capacity [17]. The specific transport of macromolecules comes cross the epithelium through M cells, which are specialized cells, active in the transcytosis process, a cellular specialization for transport of molecules across the mucosal barrier to the submucosal tissues. [18]. At the same time, tissue-specific activities of brush-border enzymes appear, with a low maltase and high lactase activities [14].This may partly be due to increased ingestion of amniotic fluid, as suggested by [15].

The changes of the epithelium are accompanied by modifications of the overall structure of the small intestine as well. Towards the end of the third month, intestinal crypts are formed and the two layers of muscular tunic differentiate [19,20]. Near birth, the small intestine grows very rapidly and in the last three weeks of gestation the volume relative to the weight of the animal increases by more than 70% [21].

To understand exactly what happens in the last period of gestation in the small intestine, we should focus on cellular turnover pathway.

**Figure 2:**
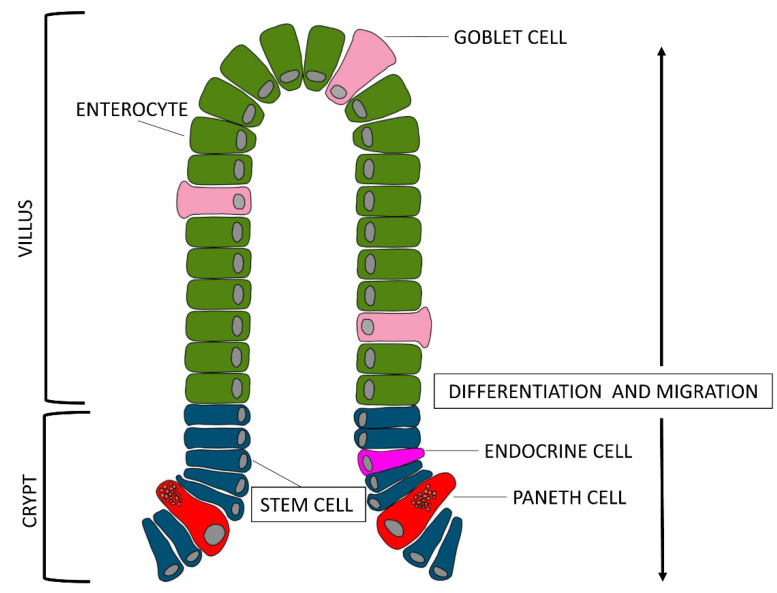
After stem cell mitosis (**blue cells**), some epithelial cells go towards the apex of the villi and differentiate into enterocytes (**green cells**) or mucous cells (**light pink cells**); some others migrate down the crypts and differentiate into Paneth cells (**red cells**) or endocrine cells (**pink cell**).

After stem cell mitosis, the fate of epithelial cells depends on the way they migrate: most of the cells go towards the apex of the villi and differentiate into enterocytes, mucous cells, or endocrine cells; some other cells migrate down the crypts and differentiate into Paneth cells (Figure 2). Enterocytes have a rapid turnover of about 48–72 h that alternates mitosis and apoptosis: the latter, a programmed physiological death, enables the removal of cells when these are old, damaged or mutated during different physiological stages, such as organogenesis, development, regeneration, and involution [22]. The dynamic balance between mitosis and apoptosis allows to maintain the correct physiology of the tissues and, if necessary, the rapid reconstruction of damaged tissues in case of pathologies [23].

Considering this, we observe that the cellular turnover in structural pattern of the gut follows a pretty precise pattern: proliferation in the crypts and migration of enterocytes and goblet cells towards the apex of the villi, where the turnover will occur with cell loss in the intestinal lumen due to apoptosis, while the endocrine cells will follow the path of the depth of the crypts. Such organized turnover allows to maintain a constant number of cells despite the speed with which this process occurs [24].

Starting from the last 3 weeks of gestation, there is a significant increase in the number of mitoses and, at the same time, a reduction in apoptosis which leads to a considerable increase in visceral mass [25,26]. Interestingly, the apoptotic cells are not localized only at the apex of the villi, but are also found along its entire axis and death occurs with detachment of groups of cells and not of single elements. In references [23,27], the authors hypothesized an important role of autophagy starting from the intrauterine pathway for the development of the intestine. Autophagy is an important cellular mechanism for the renewal of cellular components damaged following stress. It is a catabolic process targeted at recycling cellular parts or damaged organelles through a lysosome-dependent degradation pathway [28].

Basal autophagy is necessary for normal cell turnover and it is important for maintaining homeostasis, while excessive or uncontrolled autophagy promotes cell death and morbidity [29]. The mechanism of autophagy in the various intestinal cell compartments has been extensively reviewed by [30]: it is regulated by cathepsins and acts through the formation of autophagolysosomes for the degradation of cellular organelles without any change at the nuclear level [31,32].

## 4. The Development of the Small Intestine during Early Postnatal Period

After birth, morphological aspect of the enterocytes reveals the presence of the previously mentioned cytoplasmic vacuoles in fetus. Digestive vacuoles are localized in the ileum limited to the first 3–4 weeks of life, i.e., in the period in which the enzymes present in the gastric, pancreatic, and intestinal areas (lactase, aminopeptidase A and N, dipeptidase IV) are insufficient [23]. The secretion of trypsin by pancreas is, in fact, is low before weaning and increases after weaning [33]. Moreover, [34,35] also revealed an increased amylase activity from birth to 4 weeks of age, and a reduction in the activity of amylase after weaning.

Vacuoles disappear with the maturation of the intestine [16]. Fetal-type enterocytes are gradually replaced by new adult-type cells with markedly reduced endocytic activity. Furthermore, a marked increase in expression of the brush border disaccharides with a decreased lactase, and increased sucrase/maltase activities are observed in the same period of growth [36].

In the early postnatal period, structural and functional changes may in part be diet dependent [37,38]. The ingestion of colostrum causes a strong boost on the growth of the intestine, it doubles its weight and increases the length by 30% within three days of birth [39]. At birth, the small intestine in its entireness is about 2 m long and has a capacity of about 70 mL [3].

Villus length and width, crypt depth, and mucosa thickness increase from day 0 to day 3, and decreases from day 3 to day 14. Moreover, villus density reduces from day 0 to day 14 in duodenum, and from day 0 to day 3 in jejunum, Finally the surface of absorptive area increases almost 3-fold in the duodenum from day 3 to day 7 and more than 2-fold in the jejunum in the same period [40].

Reference [41] suggests that growth is associated with the stimulus that milk proteins operate on the mucosal wall and it causes an increase in cell proliferation, preceded by increased DNA replication. As already stated, in the intestinal crypts, there are stem cells that represent a center of replication and differentiation of the cells that organize the mucosa. These stem cells have the characteristic of a high mitotic replication rate: jejunum is the intestinal tract in which the cells undergo the highest rate of mitosis (40–50% of cells) starting from the second day after birth [27].

In physiological conditions, in newborn piglets, the apoptotic process affects all the cells that line the villi, while in an adult animal it is localized only at the apex of the villi. The ways in which these dead cells are removed towards the lumen involves the detachment of single cells, groups or even the entire villus. In some cases, it is possible that the cells in apoptosis migrate to the deep regions of the *lamina propria* where they are phagocytosed in the form of apoptotic bodies.

Apoptosis may be regulated in the intestinal epithelium by a reduction or an increase of apoptotic proteins from Bcl-2 family, which are proteins determining the commitment of cells to apoptosis increasing production of free radicals. Bcl-2 family proteins make enterocytes more or less sensitive to death signal mediated by other cytokines such as TNFα, which is an inducer of receptor pathway of apoptosis. As TNFα, another cytokine, the TGF-β1, is highly expressed in the very few days of life, which participates as a strong inducer of death in the epithelial cells [42].

Apoptosis in the intestinal epithelium of newborn piglets are possibly regulated by auto and paracrine factors [27]. During the first two days of life, the intestinal mucosa grows very quickly but it is still immature. In fact, it has limited capacity for its own production of functional factors and it acquires them only from colostrum or milk as external biological factors [43].

During the first two days after birth, the rate of mitotic cells temporarily increases and, consequently, a significant reduction in the rate of apoptosis is observed: this fact may explain the fast growth of the epithelium in this stage of development. Under physiological conditions, the increase in mitosis leads to an increase in the absorptive surface of the mucosa, which stabilizes until the weaning period, when a new important mitotic thrust is observed [27]. This fact is caused by the activity that solid foods apply on the cells: some nutrients in the feed perform as pro-mitotic actions. The mitosis/apoptosis ratio is an excellent indicator of intestinal growth: during the first 24 h of life, the rate of mitosis is very high due to the encrustation that milk proteins put on the intestinal mucosa. Otherwise, during weaning, the ratio does not change even if an increase in mitosis is observed, which is necessary to ensure remodeling of the intestine [23]. The remodeling of the intestinal epithelium occurs through increased proliferation in the Lieberkühn crypts. It must be emphasized, however, that the greater proliferation of stem cells in the crypts increases the possibility of mutations occurring due to replication errors and the frequency of mutation with consequent arrest of the cell cycle. If this phenomenon happened, then apoptosis would be activated by the protein p53 and by macrophages, triggered by the formation of phagocytosed apoptotic bodies, with release of the cytokine TGF-β1 [23,42].

## 5. Modifications of the Piglets Small Intestine Structure during Weaning

The weaning period is a critical phase in the development of the intestine of piglets and normally occurs between 3 and 4 weeks of life, when the animals are still feeding milk. Weaning can be considered a multifactorial syndrome caused by social and environmental stressors such as mixing of different litters, transport of animals and, of course, changes in diet [6]. Stress affects the live weight gain of animals and metabolic changes that are strongly related to the adaptation of the endocrine system.

After weaning, intestinal environment changes drastically due to a replacement of highly digestible sow milk by solid feed, mainly of plant origin. This may lead to temporary underfeeding and, consequently, undernutrition. The gastrointestinal tract has to adapt to the new type of feed, which leads to changes in gut motility, gut morphology, enzymes secretion and activity, and the composition of bacterial flora [40,41,42,43,44] studied the spatial–temporal sequence of events regarding the morphology, physiology, and ecology of the gut of piglets during the 2 weeks after weaning. The results showed that the time-dependent changes induced in the gut by weaning, can be divided into two periods: an acute period happening immediately after weaning followed, after day 5, by an adaptive and maturational phase.

Animals failing to adapt to these changes have smaller live weight gains, suffer from diarrhea, and even die because of the intestinal bacteria overgrowth [45].

Profound changes in histological parameters and enzymatic activity of the small intestine of piglets are associated with the weaning period, such as the atrophy of the villi and consequent restorative hypertrophy of the crypts. Villus atrophy after weaning is caused by an increased rate of cell loss (apoptosis), as well as a reduced rate of cell renewal (mitosis), which is highly pronounced in the duodenum when compared to the ileum [46]. At the same time, [47] observed that the tissue recovery was more pronounced in the same intestinal site starting from 3 to 14 days post weaning, thus suggesting a higher cellular turner in the proximal small intestine.

Recently, [48] proposed a new mechanism of action in the recovery of the gut tissue after weaning. The authors observed that autophagy occurs also in early weaned piglets and that inhibition of autophagy by chloroquine (CQ), an autophagy-modifying agent, regulates the process and produce beneficial effects on intestinal health and growth. Even when partial, the inhibition of the autophagy can help weaned piglets maintain intestinal mucosal homeostasis by improving intestinal morphology and barrier function, and decreasing pro-inflammatory cytokines, which produces better antioxidant status. These results provide new references for the regulation of ameliorating early weaning stress in piglets.

Finally, as already mentioned, enzymes also have a reduced activity, especially the pancreatic ones [34]: this fact results in a reduction in the digestibility of nutrients within the first week after weaning [49]. This decrease is not only linked to the quantity, but above all to the quality of pancreatic enzymes: fetal hydrolases are replaced by new isoforms and new hydrolytic enzymes such as protease E and elastase I appear, which did not exist before weaning [33]. The activity of lipase decreases [34,50] due to the lower fat content in the weaning diet than in sow’s milk and the low feed intake, usually observed, in the early post-weaning period probably due to a depletion of the enzyme stores caused by a higher rate of secretion than synthesis of enzymes [51]. At the same time, the amylase activity increased [34,35], which is probably caused by the need for amylase to digest dietary starches.

The animal takes about two weeks to restore the level and activity of the enzymes, even if the duration is indicative, as it depends on how much the animals eat and the type of protein present in the feed.

## 6. Modifications of the Gut Barrier: Role of Microflora

After birth, the piglet’s gut ecosystems undergoes a strong variation, from a sterile state to extreme colonization, with a development of a microbial community, affecting the piglet’s intestinal function and health [52]. Literature reported that the microbiota composition of a piglet’s GIT is quite stable in the suckling period, but after weaning bacteria abundance is subject to a rapid boost [53]. Dietary technique and rearing environment significantly modify the intestinal microbial community. Moreover, environmental and feed antigens represent a strong stimulus for the immune system development.

It is well known that GIT microbiota plays a key role in nutrient availability and production of short chain fatty acids (SCFAs) and vitamins, improving the defense against pathogenic bacteria.

The first line of chemical–physical defense against pathogenic antigens is represented by mucus as recently reviewed by [6]. The presence of mucus in the early postnatal period is of particular importance for the innate barrier, when animals are more sensitive to pathogens as the immune system is still being developed [54]. Mucus has a primary role in the intestinal barrier integrity, affecting its permeability as it prevents the adhesion of pathogenic bacteria through carbohydrate chains of the mucins and create a favorable environment for symbiont microflora [55]. Mucins are classified into neutral and acidic subtypes and the predominant subtype expressed in the intestinal epithelium is the acidic mucins. Researchers in [56] observed that ileum has a greater amount of acidic mucins than the other two tracts of the small intestine, suggesting it as the most susceptible region to this process. As demonstrated in rat, mouse, human, and pig, the ratio of neutral to acidic mucins generally increases between birth and the weaning period, and decreases after weaning [54].

Modifications in intestinal mucins at weaning happen due to change of feed composition, switching from highly digestible milk to less digestible, more-complex solid feed, that affect the intestinal microbiota of piglets. Moreover, the enzymes are not yet available for digestion of carbohydrates and some proteins that arrive in the large intestine are fermented by the microorganisms. Fermentation of dietary carbohydrates and protein in the pig intestine can potentially have a useful or detrimental effect on gut health [57]. The product of the fiber fermentation, the short-chain fatty acids (SCFA) represent the basal energy source for the intestinal epithelium. In particular, butyrate stimulates the turnover of epithelial cells in the ileum and large intestine. SCFAs are also able to lower the pH of the luminal environment by favoring the absorption of minerals and preventing the development of bacteria sensitive to acidic environments [58]. The composition of SCFAs depends on the type of bacteria and the substrate used for fermentation: for example, in suckling piglets, the bacterial flora mainly produces acetic acid from milk, while the casein-based milk substitutes they are substrates for butyric and propionic acid [17]. It is reported that colonic SCFA, which are distant from small intestine mucosa, stimulates cell proliferation and growth of small intestine and is likely mediated by a systemic mediatory mechanism [59]. Moreover, oligosaccharides, which represent the third most abundant components in sows’ milk and are not digested by host enzymes, have prebiotic activity that support the establishment of healthful intestinal environment of pre-weaning piglets [60].

Intestinal microbiota plays also an important role in the cell turnover: they can induce apoptosis at the epithelial level in two ways: (1) production of toxic metabolites [61]; (2) induction of inflammation, which increases the infiltration of intraepithelial lymphocytes [62].

The intestinal microbiota is therefore fundamental for the development of the mucosal epithelium and barrier properties, affecting productive parameters and health.

## 7. Conclusions

Much work has been done over the past 15 years to find solutions to minimize GIT problems in piglets throughout feeding. The need to find nutritional strategies to ensure excellent quantitative and qualitative production performance in compliance with European and national regulations is certainly pressing particularly with a view to promoting sustainable development for the farmer. In order to carry out the correct nutritional interventions, it is very important that the nutritionist is aware of the structure of the intestine and is also conscious of the most delicate stages of development in this period of the animals’ life, thus reducing antimicrobial use. In this way, the nutritional intervention becomes more precise both from the point of view of the mechanism of action of the nutrients used, and also because of the period of the animal’s life during which it is better to intervene.

Preserving the integrity of the epithelial barrier by regulating the rate of cell death is considered crucial for maintaining the intestinal homeostasis. Indeed, the functioning of the intestine during each stage of development is often evaluated based on changes in the structure of the mucosa and enzymatic activity. Proper development of the gastrointestinal tract is of significant importance for animal production. The degree of intestinal maturity affects the digestibility of nutrients, feed efficiency, and resistance to disease. It can, therefore, be important for animal performance results but the question is: does the success of weaning lie in the gut?

## Data Availability

Not applicable.
